# An Insight Into the Phytochemical Composition, Cardioprotective, and Antioxidant Characteristics of Small Knotweed (*Polygonum plebeium* R. Br.) Extract and Its Derived Fractions

**DOI:** 10.1002/fsn3.4750

**Published:** 2025-01-12

**Authors:** Muhammad Ibrar, Mir Azam Khan, Abdullah Khan, Muhammad Asghar Khan, Muhammad Saeed Jan, Abdur Rauf, Anees Ahmed Khalil, Ahood Khalid, Samiah Shahid, Mohammed Mansour Quradha

**Affiliations:** ^1^ Department of Pharmacy Bacha Khan University Charsadda Khyber Pakhtunkhwa Pakistan; ^2^ Department of Pharmacy, Faculty of Biological Sciences University of Malakand Chakdara Khyber Pakhtunkhwa Pakistan; ^3^ Department of Chemistry University of Swabi Swabi Khyber Pakhtunkhwa Pakistan; ^4^ University Institute of Diet and Nutritional Sciences, Faculty of Allied Health Sciences The University of Lahore Lahore Pakistan; ^5^ Institute of Molecular Biology and Biotechnology (IMBB), Research Centre for Health Sciences (RCHS) The University of Lahore Lahore Pakistan; ^6^ College of Education Seiyun University Seiyun Hadhramawt Yemen; ^7^ Pharmacy Department, Medical Sciences Aljanad University for Science and Technology Taiz Yemen

**Keywords:** antioxidant properties, cardioprotective, lipid lowering, phytochemicals, *Polygonum plebeium*, polyphenols

## Abstract

*Polygonum plebeium* , a member of the Polygonaceae family, is commonly known as small knotweed and has been traditionally used to treat various ailments, including cough, gastrointestinal disorders, respiratory infections, liver disease, inflammation, dysentery, eczema and ringworms, and other skin conditions. Many studies have suggested that plants belonging to this genus possess strong cardio‐protective potentials. Rats were pre‐treated with crude methanolic extract and other fractions at a dose of 500 mg/kg followed by administration of Isoproterenol hydrochloride after 24 h for 2 days. The cardioprotective effect was determined by investigating the levels of Biomarkers responsible for myocardial infarction (MI). Among all fractions Pp.CF (chloroform fraction) exhibited a significant cardioprotective effect by decreasing the levels of ALT, AST, CPK, and LDH to 74.56 ± 1.45, 95.78 ± 2.75, 156.73 ± 1.84, and 215.55 ± 5.33 IU/L in serum. The same fraction was tested for cardio‐protective potential at a dose of 50, 100, and 250 mg/kg. Pp.CF at a dose of 250 mg/kg exhibited prominent effects and reduced levels of biomarkers responsible for MI. Further investigations confirmed that Pp.CF possesses antihyperlipidemic, membrane stabilizing, and thrombolytic potential which suggests *P. plebeium* an ideal candidate for natural product isolation which will be helpful in the management of cardiovascular problems.

## Graphical abstract

*Polygonum plebeium* , known as small knotweed, has been traditionally used to treat various ailments and is now gaining attention for its cardioprotective effects. Research indicates that the chloroform fraction (Pp.CF) of this plant significantly reduces biomarkers for myocardial infarction and exhibits antihyperlipidemic, membrane stabilizing, and thrombolytic activities, making it a potential natural remedy for cardiovascular issues.

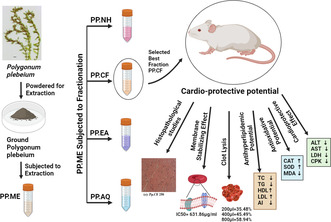

**Abbreviations**
AI: atherogenic index
ALT: alanine aminotransferase
AST: aspartate aminotransferase
CAT: catalase
CN: cellular necrosis
CPK: creatine phosphokinase
CV: central venule
CVDs: cardiovascular disease
HDL: high‐density lipoprotein cholesterol
HN: hepatocellular necrosis
INF: cellular infiltration
ISO: isoproterenol hydrochloride
LDH: lactate dehydrogenase
LDL‐C: low‐density lipoprotein cholesterol
LPO: lipid per oxidation
MDA: malondialdehyde
MI: myocardial infarction
NH: normal hepatocytes

*P. plebeium*

:
*Polygonum plebeium*

Pp.BT: butanol
Pp.CF: chloroform extract
Pp.EA: ethyl acetate extract
Pp.ME: methanolic extract
Pp.NH: N‐hexane extract
ROS: reactive oxygen species
SOD: superoxide dismutase
SS: sinosiods
TBARs: thiobarbituric acid reactive substances
TC: total cholesterol
TG: triglycerides
VLDL: very low‐density lipoprotein cholesterol level

## 1 Introduction

Cardiovascular disease (CVDs) is the leading cause of mortality throughout the word, caused more than 17.9 million deaths in 2019 (Khaltaev and Axelrod 2022). CVDs include various conditions affecting the heart and blood vessels, such as coronary heart disease, heart failure, and stroke. Risk factors for CVD include smoking, physical inactivity, unhealthy diet, high blood pressure, high cholesterol levels, diabetes and genetic interactions (Perera, Perera, et al. 2021). (Bhattarai et al. 2022). The development of myocardial infarction (MI) may involve coronary atherosclerosis and inflammation in the vascular wall. In addition to the mechanisms that mediate coronary artery occlusion and supply–demand mismatch, reactive oxygen species (ROS) are known to play a critical role in the tissue injury that follows MI (Thygesen et al. 2007). Both clinical and experimental studies have demonstrated that heart failure is associated with an elevated level of ROS, including superoxide anion (•O2−) and hydroxyl radicals (•OH). These free radicals are known to be a major factor in the lipid peroxides formation, cell membrane damage, and the destruction of antioxidative defense systems (Gayathri et al. 2011).

The administration of isoproterenol hydrochloride (ISO) to animals can induce an necrosis of myocardium and dysfunction that increases blood pressure, heart rate and bring changes in electrocardiogram (Thygesen et al. 2007), similar to effects of MI in human patients. The use of a rat model of ISO‐induced MI provides a reliable and non‐invasive approach for investigating the potential cardioprotective effects of various agents (Korkmaz et al. 2009). The most prominent factor ISO‐induced cardiac injury is the generation of free radicals through catecholamine auto‐oxidation which are highly reactive and damage cardiac cells (Afroz et al. 2016). When these free radicals attack polyunsaturated fatty acids of the cell membrane, per hydroxyl radicals are produced (Neri et al. 2015) which cause damage of fatty acids, leading to lipid per oxidation reaction (LPO), with malondialdehyde (MDA) being the end product. The loss of cell membrane integrity due to free radical attack leads to leakage of cell membrane and finally death. The level of MDA increases with increase in lipid peroxidation and the antioxidant enzymes level decreases meantime (Abhilash et al. 2011). The antioxidant defense system failure results in the accumulation of free radicals, causing oxidative stress, and eventually leading to cardiac cells apoptosis (Ibrar et al. 2019; Hazini et al. 2015). The pathophysiological abnormalities observed in the hearts of rats with cardiac necrosis are same in human MI patients.

Pharmacological interventions for CVDs include antiplatelet agents, anticoagulants, and lipid‐lowering drugs, among others. However, these treatments are associated with side effects and may not be suitable for all patients. Therefore, there is an increasing attention in the potential use of natural drugs with cardioprotective properties (Ibrar et al. 2019). MI is still the leading mortality factor worldwide, despite advancements in clinical care, increased public awareness, and the widespread use of health innovations (Sachdeva et al. 2023).

*P. plebeium* , commonly known as small knotweed, is a member of the Polygonaceae family and has been used for centuries in traditional medicine for various purposes (Nayak et al. 2024). Recently, there has been growing interest in the potential cardioprotective effects of this plant. *P. plebeium* has been traditionally used to treat various ailments, including cough, gastrointestinal disorders, respiratory infections, liver disease, inflammation, dysentery, eczema and ringworms and other skin conditions (Liao et al. 2013; Ahmad et al. 2009). Plants of this genus have been demonstrated to have remarkable heart‐protective effects in recent research studies (Liao et al. 2013; Ding et al. 2014). Literature reveals that Polygonum species are rich source of triterpenes, lignans, anthraquinones, tannins, stilbenoids, flavonoids, and phenolic acids. The cardio‐protective, antihypertensive, and dislipidemic potentials associated with various species of Polygonaceae family are due to the presence of these bioactive compounds (Khan et al. 2022). There is mounting evidence that *P. plebeium and other species of* Polygonum family has cardioprotective benefits (Liao et al. 2013) (Nayak et al. 2024). However, more study is required to determine the best dose and to understand the mechanism involved in the cardioprotection. The purpose of this study is to comprehensively explore the possible molecular targets and mechanisms of *P. plebeium* in both treatment and prevention of MI.

## 2 Materials and Method

### 2.1 Chemicals and Reagents

The following chemicals and reagents were used: Poloxamer 407 (P‐407) and Isoproterenol hydrochloride (purchased from Sigma‐Aldrich, Karachi, Pakistan) were used to induce hyperlipidemia and myocardial infarction, respectively. Simvastatin tablets (40 mg, Searle Pakistan private Limited, Karachi—Pakistan) were obtained from the market. All solvents and chemicals used were of analytical grade. The biochemical kits used to measure the lipid profiles of the rats were obtained from Merck, Karachi, Pakistan.

### 2.2 Plant Collection and Extraction

*P. plebeium* was collected from Dir Lower, 35°10′ N 72°00′ E in the month of July, shade dried and ground into a coarse powder using a cutter mill. The powder was kept in 80% methanol for 22 days while being shaken daily. The resulting mixture was filtered using a rotary evaporator, which yielded a semi‐solid methanolic extract (Pp.ME). This extract was subjected to further fractionation to obtain the N‐hexane (Pp. NH), chloroform (Pp.CF), ethyl acetate (Pp.EA), and butanol (Pp.BT) fractions.

### 2.3 Phytochemical Screening

The Pp.ME extract was subjected to phytochemical investigation to check the presence of different secondary metabolites for example, saponins, tannins, phenol, flavonoids, carbohydrates, protein, alkaloids, oils and fats, and Mucilage. These compounds are considered to be responsible for various biological activities (Ahsan et al. 2021).

#### 2.3.1 Saponins

Frothing test was used for determining the presence of saponins in Pp.ME extract. For this purpose, the representative extract was thoroughly mixed with 5 mL of distilled water in a test tube. Appearance of froth/foam indicated presence of saponins in the extract.

#### 2.3.2 Tannins

For this test, distilled water (10 mL) was mixed with Pp.ME extract and later filtered. After filtration, filtrate was thoroughly mixed with FeCl_3_ (ferric chloride) reagent. Appearance of dark blue color indicated the presence of tannins in the extract.

#### 2.3.3 Phenols

In this test, initially, Pp.ME extract was mixed with distilled water to which ferric chloride (10%) was added later. Appearance of greenish color suggests the presence of phenols in the extract.

#### 2.3.4 Flavonoids

In this analysis, Pp.ME extract was mixed thoroughly with NAOH (2 N) and appearance of yellow‐orange color indicated the presence of flavonoids in the extract.

#### 2.3.5 Carbohydrates

To assess the presence of carbohydrates in the Pp.ME extract, it was mixed with conc. H_2_SO_4_ (few drops) and Molisch's reagent (1 mL). Upon mixing, appearance of purple color suggests the presence of carbohydrates in the extract.

#### 2.3.6 Proteins

To determine the presence of proteins in the Pp.ME extract, it was mixed with Biuret reagent. Upon mixing, appearance of purple color suggests the presence of proteins in the extract.

#### 2.3.7 Alkaloids

For this test, Meyer's reagent was mixed with Pp.ME extract (1 mL). Formation of creamy‐white precipitates indicated the presence of alkaloids in the extract.

#### 2.3.8 Oils and Fats

In this test, Pp.ME extract (1 mL) was mixed with alcoholic potassium hydroxide solution (0.5 N; 1 mL) followed by addition of few drops of phenolphthalein. Resultant mixture was subjected to water bath for heating of 1–2 h. Soap formation or partial neutralization indicated the presence of oils and fats in extract.

#### 2.3.9 Mucilage

Pp.ME extract was mixed with distilled water (10 mL) followed by addition of absolute alcohol (25 mL) along with continuous stirring. Appearance of white‐cloudy precipitates indicated the presence of Mucilage.

#### 2.3.10 Determination of Total Phenolic Contents

The total phenolic content was measured using the technique described previously (Dissanayake, Bandaranayake, et al. 2022). Briefly, 1 mL of diluted extract was mixed with 9 mL of distilled water; 1 mL of Folin–Ciocalteu's reagent (FCR) was added while vigorously shaking. After 5 min, 10 mL of 7% Na_2_CO_3_ solution was added to it and thoroughly mixed. Distilled water was used to dilute the mixture to 25 mL, and it was thoroughly mixed. After 90 min, the absorbance was measured using a UV spectrophotometer at 750 nm. The total phenolic content was measured using a gallic acid standard curve. The total phenolic contents were calculated as milligrams of gallic acid equivalent (mg GAE/g) per gram of dry sample.

#### 2.3.11 Determination of Total Flavonoids Contents

The total flavonoid content of *P. plebium* was determined using the previously reported technique (Dissanayake, Bandaranayake, et al. 2022). In total, 10 mL test tube was filled with 0.3 mL of plant sample solution, 0.15 mL NaNO2 (0.5 M), 3.4 mL methanol (30%), and 0.15 mL AlCl3.6H2O (0.3 M). After 5 min, 1 cc of NaOH (1.0 M) was added to it. The absorbance of the mixture was measured using a UV spectrophotometer at 506 nm. To determine total flavonoids, a standard Rutin solution curve (0–100 mg/L) was employed. The total flavonoid concentration was reported as milligram of rutin equivalent per gram of dry sample.

### 2.4 Acute Toxicity Studies

The acute toxicity study involved dividing the animals into four groups, each consisting of six rats. The Pp.ME extract was administered orally to determine its toxicity, with the extract suspension being prepared in water and administered at doses of 1, 2, 3, and 4 g/kg. All rats were closely monitored for any signs of toxicity every half hour to 6 h on the first day, followed by daily monitoring for the next 7 days. The mortality rate was recorded for each group (Sivakumar and Rajeshkumar 2014). All the experimental protocols were approved by Ethical Research Committee (No. DREC/20160503‐18), Department of Pharmacy, University of Malakand, as per laws 2008 and Institutional Review Board of Dow University of Health Sciences with the protocol number: IRB‐864/DUHS/Approval/2017/51 for human studies.

### 2.5 In Vivo Study

Rats of either sex were procured and acclimatized for 1 week to laboratory environment in animal room of Department of Pharmacy, University of Malakand, under standard circumstances. They had free access to required pellet diet and water ad libitum. Initially, all fractions were investigated for their cardioprotective potential at 500 mg/kg dose. The most active fraction was then selected for further studies at different doses to evaluate the mechanism involved in cardio‐protection.

#### 2.5.1 In Vivo Study Details for Cardio Protective Activity

For the cardio protective activity experiment, rats were distributed into different groups, each comprising of six animals. Group 1 served as the normal group, Group 2 was used as negative control, while Groups 3, 4, and 5 received dose of 50, 100, and 250 mg/kg of Pp.CF extract, respectively. Group 6 received the Propranolol (standard drug) for 15 days at a dose of 10 mg/kg body weight. On Day 16 and 17, all animals were administered ISO subcutaneously at a dose of 150 mg/kg. Group 1 was left untreated. At the end of the experimental period, all the animals were anesthetized with diethyl ether; Blood samples were aspirated from the left ventricle after 24 h of the last ISO administration for determining the levels of ALT, AST, LDH, and CPK, and the heart was removed for histopathological studies.

#### 2.5.2 Morphological Study of Heart

The hearts of each rat were removed for histopathological studies (Goyal et al. 2015). The isolated hearts were kept in formalin solution (10%) and placed in paraffin wax. They were cut to obtain a thickness of 5 μm and stained with hematoxylin and eosin. The stained portions were studied for the detection of histopathological changes under a light microscope (Nikon, Tokyo, Japan).

#### 2.5.3 Induction of Hyperlipidemia

Hyperlipidemia in the mice was produced by intraperitoneal injection (*i.p*) of 400 mg/kg of (P‐407), which had been prepared by dissolving it in cooled distilled water (30% w/w). The solution was refrigerated overnight to ensure complete dissolution. To study the antihyperlipidemic effects, a slightly modified version (Goyal et al. 2015), and the mice were fasted for 24 h prior to inducing hyperlipidemia.

#### 2.5.4 Experimental Animals for Anti‐Hyperlipidemia Activity

A total of 36 mice weighing between 180 and 200 g were placed into different groups (*n =* 6).

Group 1, the comparison group, and the mice in this group were provided with a Regular diet having a proper amount of protein, carbohydrates, fat, fibers and vitamins. In Groups 2, 3, and 4 were administered with *i.p* injection of P‐407, followed by oral administration of 75, 150, and 300 mg/kg dose of Pp.CF. Group 5 was the hyperlipidemic group, which were made hyperlipidemic by an *i.p*. of P‐407. Group 6 was the standard group, where a dose of Simvastatin (20 mg/kg) was administered 2 h after the intraperitoneal injection of P‐407.

#### 2.5.5 Blood Sampling

Following each experiment, diethyl ether was used for anesthetic purpose of the mice, and subsequently, blood samples were collected and incubated for 30 min and centrifuged at 4°C at 4000 rpm for 10 min. The serum was separated and used to examine the levels of triglycerides (TG), total cholesterol (TC), and high‐density lipoprotein cholesterol (HDL). The level of low‐density lipoprotein cholesterol (LDL) was determined using the formula:

$$\mathrm{LDL}=\mathrm{TC}-\mathrm{HDL}-\left(\mathrm{TG}/5\right)$$

Very low‐density lipoprotein cholesterol level was determined as TG/5. The Atherogenic index was calculated by using following formula:

AI=TC/HDL

### 2.6 Determination of Antioxidant Enzymes Activity

The hearts from all rats were isolated and centrifuged for 60 min at 15,000×*g* at 4°C to obtain homogenates, which were used to estimate the activity of catalase (CAT) and superoxide dismutase (SOD) (Beers and Sizer 1952). The results were reported as units/mg protein.

For determining the activity of SOD, heart homogenate (0.1 mL) was incubated with sodium carbonate buffer (2.8 mL, 0.05 mM) 30°C for 45 min. Subsequently, an adrenaline solution (10 μL, 9 mM) was added, and the absorbance was measured at 480 nm against a blank. The results were expressed as units/mg protein.

### 2.7 Lipid Peroxidation

To assess the ability of Pp.CF to inhibit lipid peroxidation, we measured the formation of malondialdehyde, thiobarbituric acid reactive substances by previously reported method (Ledwozyw et al. 1986). The malondialdehyde production is a reliable marker of lipid peroxidation and was measured using a Shimadzu UV–visible spectrophotometer with a maximum absorption at 535 nm.

### 2.8 Human Study

#### 2.8.1 Thrombolytic Effect

Fresh human blood was collected from sixty (60) healthy volunteers having no use of oral contraceptives, cigarette smoking and anticoagulant (Ibrar et al. 2019). Blood sample (1 mL) were distributed in pre‐weighted Eppendorf tubes (*n* = 6) and incubated for 90 min at 37°C for clot formation. After formation of clot, the serum was completely removed without disturbing the clot. The tubes were again weighted to determine the clot weight. Pp.CF was mixed with distilled water and kept overnight at room temperature. The soluble portion of the test sample was separated in water as supernatant and was used for thrombolytic activity. The test sample was added to different tubes containing pre‐weighted clot at a dose of 200, 400, and 800 μL. Streptokinase (100 μL) and distilled water (500 μL) was used as positive and negative control, respectively. The blood was transferred to micro centrifuge tubes and was allowed for clot formation. The serum was separated and each clot weight was determined using following formula: Clot weight = weight of tube containing clot‐weight of tube without clot.

Tubes containing pre‐weighted clot were added with the test sample at 200, 400, and 800 μL concentration and were incubated at 37°C for 90 min. The fluid was removed and the tubes were weighted again to determine the clot lysis. The percent clot lysis was calculated using the following equation:

$$\%\text{of clot lysis}=\left(\text{weight of released clot}/\text{clot weight}\right)\times 100$$

StreptoKinase (100 μL) was used as standard drug while normal saline served as non‐thrombolytic control.

#### 2.8.2 Membrane Stabilization Action

To assess the membrane stabilization potential of Pp.CF, we followed the standard protocol for hemolysis inhibition (Sadique et al. 1989). Fresh blood samples (5 mL) were received from 30 healthy participants (*n* = 3 per tested sample) who had not used non‐steroidal anti‐inflammatory drugs for 2 weeks. Erythrocytes were separated by centrifugation of the blood for 10 min at 3000 rpm, the supernatant was discarded. The obtained pellet was washed with isotonic buffer (pH 7.4, containing 154 mM NaCl in 10 mM sodium phosphate buffer) until obtaining a clear supernatant. The volume of the erythrocytes was noticed and reconstituted with isotonic buffer as a 40% v/v suspension.

Next, the suspension (50 μL) was mixed with hypotonic buffer and incubated with various concentrations of Pp.CF (100 μL) in isotonic buffer. Supernatant was obtained and absorbance was checked at 560 nm. The percent hemolysis inhibition caused by Pp.CF and standard drug was determined using the formula:

$$\begin{array}{c} \text{Percent hemolysis inhibition}=(\text{absorbance of control}-\\ \;\text{absorbance of sample}/\text{absorbance of control})\times 100 \end{array}$$

### 2.9 Statistical Analysis

Statistical analysis was performed by using SPSS version 16 (SPSS Inc. Chicago IL, USA). Data is re‐ported as a mean of six animals per group ± SEM. Statistical analyses were conducted by one way and two‐way ANOVA followed by post hoc Tukey test for multiple comparisons. All the values of *p* < 0.05 were considered significant.

## 3 Results

### 3.1 Phytochemical Screening

The qualitative phytochemical screening of crude methanolic fraction *P. plebejum* revealed the presence of saponins, tannins, phenols, carbohydrates and alkaloids, proteins, flavonoids, oils, and fats while mucilage was not detected (Table 1).

**TABLE 1:** Phytochemical screening of crude methanolic fraction of *Polygonum plebejum*.

Serial no	Secondary metabolites	Result
i	Saponins	+
ii	Tannins	+
iii	Phenol	+
iv	Flavonoids	+
v	Carbohydrates	+
vi	Protein	+
vii	Alkaloids	+
viii	Oils and fats	+
ix	Mucilage	−

Abbreviations: +, indicates presence of phytochemicals; −, indicates absence of phytochemicals.

### 3.2 Total Phenolic and Flavonoid Contents

The total phenolic and flavonoid contents in different fractions of *P. plebeium* are shown in Table 2. The results show that PP.CF, Pp.EA, and Pp.ME have high phenolic contents (81.74 ± 1.17, 66.58 ± 0.97, and 64.62 ± 0.73 mg GAE/g of dry sample, respectively). PP.CF, Pp.ME, and Pp.EA had the highest flavonoids content, with 89.20 ± 1.69, 70.47 ± 1.23, and 67.32 ± 1.73 mg RTE/g of sample, respectively.

**TABLE 2:** Total phenolics and total flavonoids contents of *Polygonum plebeium*.

Samples	Total phenolics (mg GAE/g of sample)	Total flavonoids (mg RTE/g of sample)
Pp.ME	64.62 ± 0.73	70.47 ± 1.23
Pp.NH	39.92 ± 1.07	43.53 ± 2.43
Pp.CF	81.74 ± 1.17	89.20 ± 1.69
Pp.EA	66.58 ± 0.97	67.32 ± 1.73
Pp.BT	17.88 ± 1.07	26.37 ± 1.67

### 3.3 Acute Toxicity Studies

Acute toxicity study was performed on rats using Pp.ME at different doses. The results showed that extract was safe up to a dose of 3 g/kg body weight, with no systemic toxic effects. These findings demonstrate the high biocompatibility of the test sample and confirm that use of *P. plebeium* is safe in laboratory animals in the present study, at the tested doses.

### 3.4 Cardio Protective Effect

Pp.ME and its different fractions were evaluated at a dose of 500 mg/kg for their cardioprotective activity based on their ability to reduce the levels of ALT, AST, CPK, and LDH, which increases after the administration of ISO (Table 3). Among the tested samples, Pp.CF was noted as a most effective cardioprotective agent at this dose (Table 3). To further investigate its potency, Pp.CF was tested at doses of 50, 100, and 250 mg/kg body weight. The 50 and 100 mg/kg doses did not produce significant differences in cardiac marker enzyme activity, but a marked decrease in enzyme activity was observed in the serum of the group receiving Pp.CF at 250 mg/kg body weight compared to the ISO group. Therefore, Pp.CF demonstrated a highly significant cardioprotective effect at the dose of 250 mg/kg. Specifically, the serum levels of ALT, AST, CPK, and LDH were significantly reduced in the group that received Pp.CF at 250 mg/kg dose in comparison with control group. The results are summarized in Table 4.

**TABLE 3:** Cardioprotective potential of Pp.ME and subsequent fractions at 500 mg/kg.

Samples 500 mg/kg	AST (IU/L)	ALT (IU/L)	CPK (IU/L)	LDH (IU/L)
Normal	64.45 ± 2.67	85.16 ± 2.13	146.53 + 2.26	198.83 + 3.48
ISO group	167.57 ± 3.67	182.16 ± 1.73	281.83 ± 4.56	435.5 ± 5.45
Pp.ME	122.83 ± 4.33**	140.33 ± 2.54***	185.45 ± 2.56**	261.54 ± 7.54***
Pp.NH	98.38 ± 4.08^ns^	124.35 ± 2.67***	178.50 ± 2.44*	364.80 ± 6.07***
Pp.CF	74.56 ± 1.45***	95.78 ± 2.75***	156.73 ± 1.84***	215.55 ± 5.33***
Pp.EA	103.45 ± 2.76*	108.50 ± 2.82***	200.66 ± 3.77***	343.64 ± 5.29***
Pp.AQ	138.57 ± 4.56^ns^	160.43 ± 4.02***	198.35 ± 3.73^ns^	287.37 ± 4.34***
Standard (propranolol)	72.45 ± 0.81***	93.16 ± 127***	153.14 ± 0.57***	212.85 ± 1.21***

*Note:* ***p < 0.001, **p < 0.01, *p < 0.05, ^ns^ *p* > 0.05.

**TABLE 4:** Cardioprotective potential of Pp.CF at different doses.

Groups	AST (IU/L)	ALT (IU/L)	CPK (IU/L)	LDH (IU/L)
Normal	64.45 ± 2.67	85.16 ± 2.13	146.53 + 2.26	198.83 + 3.48
ISO treated	167.57 ± 3.67	182.16 ± 1.73	281.83 ± 4.56	435.5 ± 5.45
Pp.CF (50 mg /kg)	105.33 ± 2.40***	146.35 ± 2.88***	191.33 ± 5.62***	256.16 ± 2.53***
Pp.CF (100 mg /kg)	94.66 ± 2.47***	118.83 ± 3.00***	182.83 ± 2.93***	243.65 ± 1.06***
Pp.CF (250 mg /kg)	77.96 ± 1.09***	102.33 ± 145***	165.43 ± 2.63***	220.38 ± 4.05***
Standard (10 mg /kg)	72.45 ± 0.81***	93.16 ± 127***	153.14 ± 0.57***	212.85 ± 1.21***

*Note:* ***p < 0.001, **p < 0.05, *p < 0.05, ^ns^ *p* > 0.05.

### 3.5 Antihyperlipidemic Potential

According to the results obtained, the P‐407 hyperlipidemic group had lowered the levels of HDL significantly and increased levels of TC, TG, and LDL significantly in comparison to healthy control group. However, in mice with hyperlipidemia, the Pp.CF therapy demonstrated dose‐dependent anti‐hyperlipidemic effects. Pp.CF slightly decreased the blood levels of TC, TG, and LDL in comparison to the control group at doses of 50 and 100 mg/kg (Simvastatin). The potential for decrease in hyperlipidemia enhanced with increasing the concentrations of test samples. At 250 mg/kg, Pp.CF demonstrated a considerable reduction in the TC, TG, and LDL levels, which were comparable with standard drug (Simvastatin). Moreover, the Pp.CF therapy reduced the AI to 3.49 ± 0.17 at the same concentration, which was comparable to the AI of Simvastatin (3.21 ± 0.83) Table 4. These findings imply that Pp.CF of *P. plebiuem* can successfully reduce plasma lipid levels and be used to treat MI.

### 3.6 Estimation of Antioxidant Enzymes

In a homogenate of heart tissue, the catalase (CAT) and super oxide dismutase (SOD) activities were evaluated. In Comparison to the healthy control group, the treatment with ISO subcutaneously caused a substantial decline in CAT and SOD activity. Nevertheless, CAT and SOD levels increased in a dose‐dependent manner following pre‐treatment with Pp.CF at varied doses. Moreover, compared to the ISO‐treated group, prophylactic treatment of Pp.CF at 250 mg/kg increased CAT and SOD activities significantly to 52.46 ± 1.04 mmol/g tissue and 46.37 ± 1.79 (U/g tissues), respectively (Table 5).

**TABLE 5:** Estimation of antioxidant enzymes.

Sample	CAT (mmol/g tissue)	SOD (U/g tissues)	MDA (mmol/g tissues)
Normal	63.62 ± 3.67	55.84 ± 2.83	0.82 ± 0.12
Iso treated	31.58 ± 1.87*	29.02 ± 1. 53*	3.67 ± 0.87^ns^
Pp.CF (50 mg/kg)	35.36 ± 1.69*	37.28 ± 2.01**	1.70 ± 0.64^ns^
Pp.CF (100 mg/kg)	42.93 ± 1.91**	40.70 ± 1.26**	1.25 ± 0.44^ns^
Pp.CF (250 mg/kg)	52.46 ± 1.04***	46.37 ± 1.79^***^	1.03 ± 0.23^ns^
Propranalol (10 mg/kg)	55.71 ± 1.47***	49.06 ± 2.05***	0.95 ± 0.13^ns^

*Note:* ***p < 0.001, **p < 0.05, *p < 0.05, ^ns^ *p* > 0.05.

### 3.7 Thrombolytic Potential

The thrombolytic activity of Pp.CF was tested at various concentrations. To serve as a positive control, 30,000 I.U of Streptokinase was utilized and exhibited a percent clot lysis of 79.83%. The highest thrombolytic activity of 58.94% was observed by adding 800 μL of Pp.CF to the clots. The moderate lysis of 45.49% and 35.48% was observed at a dose of 400 and 200 μL, respectively (Table 6). On the other hand, distilled water, which was used as a negative control, had only 6.35% thrombolytic activity. These effects suggest that Pp.CF possesses thrombolytic activity and could be a promising therapeutic option for MI caused by thrombosis.

**TABLE 6:** Thrombolytic effect of Pp.CF at various concentrations.

Samples	Conc. (μL)	W1	W2	W3	Thrombolytic potential (%)
Pp.CF	200	891.87 ± 0.69^ns^	927.97 ± 0.93^ns^	915.16 ± 1.02***	35.48%***
	400	892.05 ± 0.37^ns^	928.03 ± 0.83^ns^	911.66 ± 1.21***	45.49***
	800	891.93 ± 0.42^ns^	927.83 ± 0.62^ns^	906.67 ± 0.92***	58.94**
Streptokinase	100	891.67 ± 0.86	927.33 ± 1.35	901.16 ± 1.05	73.83
Dist. water	500	891.94 ± 0.70	927.89 ± 0.95	925.23 ± 0.87	7.39

*Note:* ***p < 0.001: highly significant, **p < 0.05: significant; in comparison to streptokinase.

Abbreviation: ns, not significant to control.

### 3.8 Membrane Stabilization Potential

The study investigated the membrane stabilizing effect of Pp.CF on human erythrocyte membranes. The extract was tested at different concentrations (62.5, 125, 250, 500, and 1000 μg/mL), and the percentage of membrane stabilization activity was recorded. The results showed that the Pp.CF exhibited concentration‐dependent membrane stabilizing activity, with a maximum of 59.94% ± 1.04% at 1000 μg/mL. The IC50 value of Pp.CF was 631.86 μg/mL, indicating that higher concentrations were required to achieve half‐maximal inhibition. The study also compared the membrane stabilizing activity of Pp.CF with acetyl salicylic acid, a standard drug known for its membrane stabilizing potential. The results showed that acetyl salicylic acid exhibited higher membrane stabilizing activity than Pp.CF, with a maximum of 78.45% ± 1.84% at 1000 μg/mL (Table 8). The IC_50_ value of acetyl salicylic acid was also lower (218 μg/mL) than that of Pp.CF, indicating that it was more potent.

Based on these results, the study suggests that Pp.CF has cardioprotective potential through membrane stabilization activity, although it is less potent than acetyl salicylic acid. Further studies are needed to investigate the mechanism of membrane stabilization by Pp.CF and to determine its efficacy in vivo.

### 3.9 Histopathological Study

**FIGURE 1:**
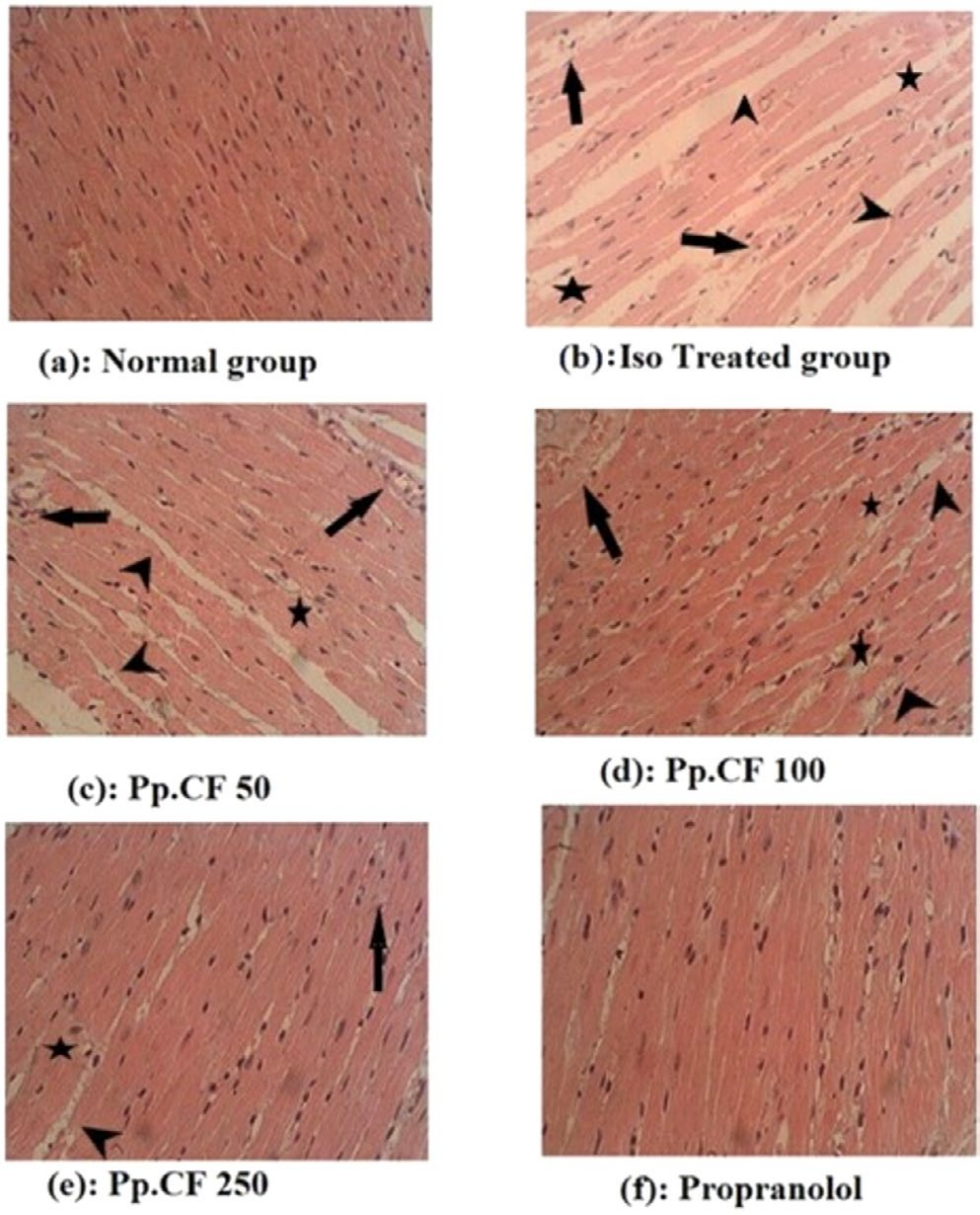
Histological findings profile. 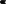
Chevron indicates myocardial damage with fragmented and separated cardiac muscle fibers. 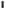
Arrow indicate the vascular leakage of RBCs, which mostly occurs during myocardial infarction. 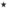
Star indicates necrosis and cellular infiltration.

The histological findings are shown in Figure 1. Normal cardiac muscle bundles were visible in the heart tissue of control rats, which showed no symptoms of inflammation 1(a). In contrast, rats given Isoproterenol hydrochloride alone showed severe inflammatory symptoms in their myocardium, such as membrane damage, cellular infiltration, and localized myonecrosis 1(b). On the other hand, the disease control group, rats pre‐treated with Pp.CF at doses of 50, 100, and 250 mg/kg body weight showed a reduction in inflammatory symptoms and myonecrosis.

## 4 Discussion

The urgent need to develop effective therapies for managing CVDs, particularly MI, stems from the fact that CVDsare the leading cause of death and morbidity globally. The identification of new herbal products and their active constituents with cardioprotective characteristics has the potential to provide beneficial benefits in avoiding cardiovascular dysfunction in MI since oxidative tissue damage and inflammation play essential roles in the pathogenic processes of MI.

The repetitive administration of ISO presents a low‐mortality, non‐invasive animal model of acute myocardial injury that closely resembles acute MI in humans (Brooks and Conrad 2009). Thus, the ISO‐induced myocardial damage experimental model has been utilized to evaluate the impact of different treatments on cardiac dysfunctions. According to our investigation, rats administered with ISO had considerably higher ALT, AST, CPK, and LDH serum levels in comparison with control group (Table 2). Along with this, there were numerous neutrophil infiltrations and morphological abnormalities such necrotic characteristics and misaligned myofibrils. Those alterations are suggestive of myocyte apoptosis and necrosis, which are frequently preceded by membrane breakdown and subsequently followed by the release of cardiac biomarkers into the bloodstream. The circulation levels of these biomarkers therefore rise (Salehi et al. 2018). The serum levels of ALT, AST, CPK, and LDH were significantly reduced in the group that received Pp.CF at 250 mg/kg dose in comparison with control group. It Significantly decreased the levels of ALT, AST, CPK, and LDH to 77.96 ± 1.09, 102.33 ± 145, 165.43 ± 2.63, and 220.38 ± 4.05 IU/L, respectively, indicating the ameliorative potential of the plant in ISO induced rats (Table 3) which may be correlated with previous study that investigated the effect *Rumex vesicarius* on biomarkers responsible for MI. (Khan et al. 2022).

The primary risk factor for heart diseases, including atherosclerosis, stroke, and death, is hyperlipidemia. This condition is often caused by a diet that is high in fat, which can lead to the development of both hyperlipidemia and atherosclerosis. Over time, these conditions can result in coronary heart disease (CHD) (Sheneni et al. 2018). Elevation in TG, TC, and LDL were observed in animals treated with P‐407. Animals treated with Pp.CF 250 mg/kg reduced the TG, TC, and LDL levels significantly. The elevation in TC concentration is due to indirect stimulation of HMG‐CoA reductase via an intraperitoneal injection of P407 (Johnston, Punjabi, and Froelich 1992). Therefore, any potential TC‐lowering effects observed with Pp.CF 250 mg/kg treatment may be attributed to a reduction in hepatic HMG‐CoA reductase activity and/or an increase in the activity of cholesterol‐7‐alpha‐hydroxylase, the enzyme responsible for converting cholesterol into bile acids. The elevation in TG level observed following P407 i.p. administration is mainly due to the inhibition of TG degradation. This is because capillary lipoprotein lipase is directly inhibited by P407, which is responsible for hydrolysis of TG present in plasma. While the standard drug may not decrease TAG concentrations by activating lipoprotein lipase, the administration of Pp.CF 250 mg/kg has been shown to reduce TAG levels through multiple mechanisms. It includes activating endothelium‐bound lipoprotein lipase, which hydrolyzes triglycerides into fatty acids, as well as inhibiting lipolysis to prevent fatty acids from being converted back into triglycerides. This study has demonstrated a significant (*p* < 0.001) reduction in LDL levels following treatment with Pp.CF 250 mg/kg. At 250 mg/kg, Pp.CF demonstrated a considerable reduction in the TC, TG, and LDL levels to 114.42 ± 3.03***, 121.50 ± 4.34***, and 58.43 ± 2.67 mg/dL which were comparable with standard drug (Table 4). The phytochemical evaluationof *P. plebeium* shows the presence of phenols and this finding is consistent with previous research (Sheneni et al. 2018), which suggested that phenolics could increase LDL receptor densities in the liver, resulting in greater binding to apolipoprotein B and thereby enhancing the liver's ability to remove LDL from the bloodstream.

Oxidative stress is widely recognized as a major contributor to the pathogenesis of MI. As such, it is logical to expect that antioxidant treatments would unregulate intracellular antioxidant enzymes and preventing reperfusion injury. Indeed, recent research has shown that the administration of edaravone, a free radical scavenger, to MI patients results in a significant reduction in infarct size and reperfusion arrhythmia (Johnston, Punjabi, and Froelich 1992; Tang et al. 2006). In current study pre‐treatment of Pp.CF at 250 mg/kg increased CAT and SOD activities significantly to 52.46 ± 1.04 mmol/g tissue and 46.37 ± 1.79 (U/g tissues), respectively (Table 7). These results are in agreement with previous reported study which revealed that leaf extract of *Rumex vesicarius* increased the level of GSH whereas, decreased the level of LPO in ISO induced Mi in rats (Ahmed 2015).

**TABLE 7:** Antihyperlipidemic potential of Pp.CF in P‐407 induced hyperlipidemic mice.

Groups	TC	TG	HDL	LDL	AI
Normal	76.34 ± 2.30	94.15 ± 1.63	38.44 ± 1.33	20.55 ± 2.86	2.15 ± 0.32
P 407	208.60 ± 5.23	198.32 ± 7.32	21.33 ± 1.66	149.70 ± 5.43	11.53 ± 0.66
Simvastatin	103.63 ± 2.03*	112.86 ± 3.48**	33.82 ± 1.24^ns^	47.32 ± 2.19^ns^	3.21 ± 0.83^ns^
Pp.CF (50 mg/kg)	147.94 ± 4.33**	162.33 ± 5.13**	27.64 ± 1.73^ns^	87.72 ± 6.65**	5.23 ± 0.73^ns^
Pp.CF (100 mg/kg)	126.79 ± 3.73***	145.76 ± 5.23***	29.03 ± 1.11^ns^	67.91 ± 3.56*	4.13 ± 0.22^ns^
Pp.CF (250 mg/kg)	114.42 ± 3.03***	121.50 ± 4.34***	31.79 ± 1.11^ns^	58.43 ± 2.67***	3.49 ± 0.17^ns^

*Note:* Values of TC, TG, HDL, and LDL are presented as mg/dL. Where ***p < 0.001, **p < 0.01, ^ns^ *p* > 0.05 as compare to P 407 treated group.

Membrane destabilization is a common feature of MI, those results from the initiation of membrane lipid peroxidation by free radicals, ultimately causing damage to the structural and functional integrity of the heart tissues (Zaafan et al. 2013). In this regard, cardioprotective drugs are supposed to exert a stabilizing effect on the membrane of myocardial cells. Our current study has demonstrated that Pp.CF significantly reduces oxidative stress in comparison to the ISO group (***p < 0.001) and protects against cellular membrane damage, as evidenced by a marked reduction in membrane destabilization, which could be attributed to its polyphenolic and flavonoid contents. These polyphenolic compounds are important scavengers of free radicals, that protect cell membranes against oxidative attack (Aldini et al. 2003).

In this study, Histopathological examination of ISO‐treated rats showed myocardial necrosis, inflammatory cells infiltration, and Disruption of muscle fiber continuity in comparison to the control group. However, treatment with Pp.CF resulted in the restoration of the parameters mentioned earlier, which was similar to that of the standard (propranolol) group as earlier study reported on medicinal plant “Rheum turkestanicum” belonging to family polygonaceae decreased inflammatory cells and myocardial degeneration (Hosseini et al. 2022). The present study demonstrates that Pp.CF extract exerts cardioprotective activity through multiple mechanisms including recovery of enzyme levels, hypolipidemic effects, antioxidant activity, membrane stabilizing potential, thrombolytic effects, and Ameliorating reperfusion injury.

Our findings from the thrombolytic test have significant suggestions for cardioprotective effect. Streptokinase, extensively utilized thrombolytic drug, converts plasminogen to plasmin but has numerous adverse effects, prompting researchers to search for an alternative agent (Bhowmick et al. 2014). We investigated whether different extracts of plant possess thrombolytic property by comparing the negative and positive controls. We found that addition of water did not cause any clot lysis but the addition of different concentrations of Pp.CF resulted in significant clot lysis. The highest clot lysis was observed at a concentration of 800 μL of Pp.CF (58.94%). Previous studies have suggested that alkaloids, tannins and saponins are responsible for dissolution of blood clots activity (Uddin et al. 2020), as these phytochemicals disrupt fibrinogen and fibrin in a clot, ultimately leading to fibrinolysis. Phytochemical analysis of the crude extract revealed the presence of tannins, alkaloids, and saponins, suggesting that these phytochemicals may be responsible for the clot lysis activity observed in the Pp.CF fraction. According to a study conducted by Saleem et al. (2018), *P. plebeium* was reported to be rich source of flavonoids and constituents like gingerols, protocatechuic acid, lyoniresinol 9′‐sulfate, and gallic acid were found to be among vital biologically active phenolics. They concluded that owing to presence of these bioactive compounds, *P. plebeium* may be used as a natural source of constituents for nutraceutical industry (Saleem et al. 2018). Therefore, based on the findings of our study, it can be concluded that the chloroform extract of *P. plebeium* is a significant source of therapeutic agents having cardioprotective effects due to presence of diverse flavonoids and phenols in it.

**TABLE 8:** Membrane stabilizing potential of Pp.CF at various concentrations.

Sample	Conc. (μg/mL)	Percent hemolysis inhibition	IC_50_ (μg/mL)
Pp.CF	62.5	19.39 ± 0.95***	631.86
	125	22.65 ± 1.69***	
	250	31.00 ± 0.93***	
	500	46.30 ± 1.73**	
	1000	59.94 ± 1.04*	
Standard drug (acetyl salicylic acid)	62.5	29.53 ± 1.72	218.02
	125	34.76 ± 1.34	
	250	49.40 ± 1.42	
	500	70.08 ± 1.57	
	1000	78.45 ± 1.84	

*Note:* ***p < 0.001, **p < 0.05, *p < 0.05, ^ns^ *p* > 0.05.

## 5 Conclusion

In current research work we selected *P. plebeium* on the bases of literature survey and explored its different fractions for their cardioprotective potential. Among all fraction Pp.CF exhibited significant cardioprotective effect by decreasing the levels of biomarkers responsible for myocardial infarction. The same fraction was tested for cardioprotective potential at lower doses. Further investigations confirmed that Pp.CF possesses antihyperlipidemic, membrane stabilizing, thrombolytic potential which suggest *P. plebeium* as an ideal candidate for natural products isolation which will be helpful in the management of cardiovascular problems.

## Author Contributions

**Muhammad Ibrar:** conceptualization (lead), data curation (lead), investigation (lead), methodology (lead), validation (equal), visualization (equal), writing – original draft (lead), writing – review and editing (lead). **Mir Azam Khan:** investigation (equal), methodology (equal), validation (equal), visualization (equal), writing – original draft (equal), writing – review and editing (equal). **Abdullah Khan:** investigation (supporting), methodology (supporting), validation (equal), visualization (supporting), writing – original draft (supporting), writing – review and editing (equal). **Muhammad Asghar Khan:** investigation (equal), methodology (equal), validation (equal), visualization (equal), writing – original draft (equal), writing – review and editing (equal). **Muhammad Saeed Jan:** methodology (supporting), validation (supporting), visualization (supporting), writing – original draft (supporting), writing – review and editing (supporting). **Abdur Rauf:** investigation (equal), methodology (equal), validation (equal), visualization (equal), writing – original draft (equal), writing – review and editing (equal). **Anees Ahmed Khalil:** investigation (equal), methodology (equal), validation (equal), visualization (equal), writing – original draft (equal), writing – review and editing (equal). **Ahood Khalid:** investigation (equal), methodology (supporting), validation (supporting), visualization (supporting), writing – original draft (supporting), writing – review and editing (equal). **Samiah Shahid:** investigation (equal), methodology (equal), validation (equal), visualization (equal), writing – original draft (equal), writing – review and editing (equal). **Mohammed Mansour Quradha:** investigation (equal), methodology (equal), validation (equal), visualization (equal), writing – original draft (equal), writing – review and editing (equal).

## Conflicts of Interest

The authors declare no conflicts of interest.

## Data Availability

The required data can be provided upon reasonable request to the corresponding author.
